# Learning from the dead: improving safety while placing unconscious trauma patients in various lateral positions

**DOI:** 10.1186/1757-7241-22-S1-O4

**Published:** 2014-07-07

**Authors:** Per Kristian Hyldmo, Bryan P Conrad, Dewayne N Dubose, Jo Røislien, Mark Prasarn, Eldar Søreide, Glenn Rechtine, MaryBeth Horodyski

**Affiliations:** 1 Research Department, Norwegian Air Ambulance Foundation, Drøbak, Norway; 2 Network for Medical Sciences, University of Stavanger, Stavanger, Norway; 3 Department of Orthopaedics & Rehabilitation, University of Florida, Gainesville, Florida, USA; 4 Department of Orthopaedics, University of Texas, Huston, Texas, USA; 5 Associate Chief of Staff, Bay Pines VAHCS, St. Petersburg, Florida, USA; 6 Department of Biostatistics, University of Oslo, Oslo, Norway; 7 Department of Anaesthesiology and Intensive Care, Stavanger University Hospital, Stavanger, Norway

## Background

The unconscious trauma patient with a possible unstable spinal injury constitutes a clinical challenge. To protect the unintubated airway, some guidelines [1,2] recommend that the patient be turned into a lateral position, e.g. the Recovery Position (RP) [1] or the Lateral Trauma Position (LTP) [2]. Other lateral positions have also been proposed, as the HAINES position [3] and variations thereof. However, moving the patient may cause secondary neurological injury. The aim of this study was to explore how much motion lateral position techniques produce in an unstable cervical spine injury.

## Method

We surgically created a global ligamentous instability between C5 and C6 in five fresh cadavers [4]. Four different techniques were evaluated; RP, LTP and two varieties of HAINES (one or both legs flexed; H1 and H2). Relative angular and linear motion between C5 and C6 was measured using an electromagnetic tracking device (Liberty, Polhemus Inc.™, Colchester, VT). Each method was repeated tree times in each cadaver. Both angular and linear movements were measured. Data were analysed using generalized linear mixed models (GLMM), adjusting for intra-cadaver correlation.

## Results

Compared to RC, LTP created significantly less movement during lateral bending (p=.037), while H1and H2 had significantly less movement than RC in axial translation (p=.009 and .033). There was a tendency towards LTP and H1 and H2 performing better than RC also for other movements.

## Conclusion

Our results indicate that in unconscious trauma patients, LTP or one of the two HAINES techniques is preferable to the classic recovery position in the setting of an unstable cervical spine injury.
